# A Facile Stereoselective Total Synthesis of (*R*)-Rugulactone

**DOI:** 10.1155/2014/767954

**Published:** 2014-03-30

**Authors:** B. Narasimha Reddy, R. P. Singh

**Affiliations:** ^1^Polymer Science and Engineering Division, National Chemical Laboratory, Pashan, Pune 411 008, India; ^2^Bharati Vidyapeeth University, Advanced Research Centre in Pharmaceutical Sciences & Applied Chemistry, Poona College of Pharmacy, Erandwane, Pune 411038, India

## Abstract

An efficient and novel synthesis of (*R*)-rugulactone has been achieved employing Sharpless asymmetric epoxidation of allyl alcohols followed by selective hydride reduction of epoxy alcohols and olefin cross metathesis reactions.

## 1. Introduction

**Figure 1:**
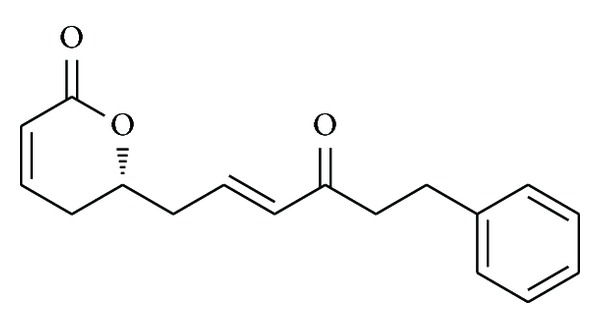
(*R*)-Rugulactone (**1**).

**Scheme 1:**
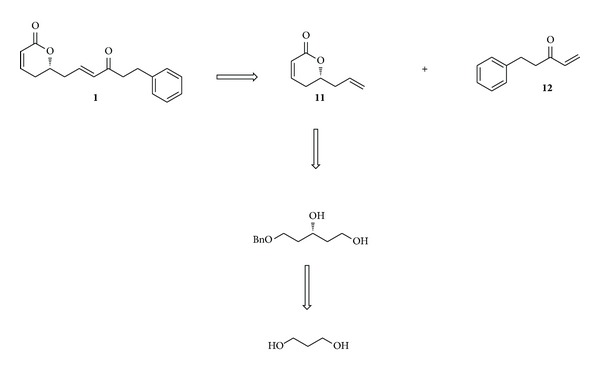
Retrosynthesis of (*R*)-rugulactone.

The 6-alkyl and aryl substituted *α*-pyrones (6-arylalkyl-5,6-dihydro-2H-pyran-2-ones) possess important biological properties such as antitumor, antiviral, antifungal, and anti-inflammatory [1–12]. These properties arise as a result of Michael acceptor property of *α*-pyrones towards the amino acid residues of the receptors. The biological assays of 6-arylalkyl-5,6-dihydro-2H-pyran-2-one, (*R*)-rugulactone (**1**), which has been extracted from the evergreen tree* Cryptocarya rugulosa *[13] of Lauraceae family, have been found to inhibit the nuclear factor (NF-*κ*B) activation pathway occurring in different types of cancers [14–18]. Due to the attractive biological activity of (*R*)-rugulactone (**1**) (Figure 1), several total syntheses have already been reported in the literature [19–25]. In those reported syntheses the chiral center was created by different means: by Jacobsen's hydrolytic kinetic resolution of epoxides [19], by Keck's asymmetric allylation [21], by proline catalyzed *α*-aminoxylation [22] of aldehydes, by enzymatic resolution of racemic homoallylic alcohols [23], and by using a chiral pool [24, 25]. In this communication, we describe the stereoselective synthesis of (*R*)-rugulactone starting from inexpensive starting materials. The Sharpless asymmetric epoxidation of allyl alcohols followed by selective hydride reduction affords 1, 3-diols with high stereoselectivity. These chiral 1, 3-diols are versatile synthetic intermediates for a variety of biologically active molecules [26–28]. The retrosynthetic strategy of our synthesis is depicted in Scheme 1, which involves Grubb's cross metathesis between compounds** 11** and** 12**.

## 2. Materials and Methods

### 2.1. General Information

Solvents were purified and dried by standard procedures before use. Optical rotations were measured using sodium D line on a JASCO-181 digital polarimeter. IR spectra were recorded on Thermo Scientific-Nicolet 380 FT-IR Instrument. ^1^H NMR and ^13^C NMR spectra were recorded on Brucker AC-200 spectrometer. Elemental analysis was carried out on a Carlo Erba CHNS-O analyzer. Full experimental details, ^1^H and ^13^C NMR spectra, can be found in Supplementary Material available online at http://dx.doi.org/10.1155/2014/767954.

### 2.2. ((3S)-3-(2-(Benzyloxy)ethyl)oxirane-2-yl)methanol, **4**

(−)-Diethyl tartarate (0.2 g, 1 mmol) and Ti(O-iPr)_4_ (0.23 g, 0.8 mmol) were added sequentially to a suspension of 4 Å molecular sieves (3 g) in CH_2_Cl_2_ (20 mL) at −20°C and the suspension was stirred for 30 min. A solution of compound** 3 **(0.6 g, 2.6 mmol) in dry CH_2_Cl_2 _(15 mL) was then added dropwise at the same temperature followed by the addition of tBuOOH (0.45 g, 2 mmol) and the reaction mixture was stirred for 12 h at −10°C. When the starting material was not observed on the TLC, the reaction was quenched with 20% NaOH solution saturated with NaCl (1 mL) and the reaction mixture was stirred vigorously for another 30 min at RT. The resulting reaction mixture was filtered through Celite, the solvent was evaporated, and the crude product was purified by column chromatography over silica gel (60–120 mesh, EtOAc/hexane 3 : 7) to afford pure epoxy alcohol** 4** in 87% yield (0.54 g);[α]D25: + 16.9 (*c* 0.6, CHCl_3_); IR (neat): *ν* 3478, 3125, 3053, 2920, 1585, 1267, 1250, 1192, 1124, 1094, 845, 790, 744 cm^−1^; ^1^H NMR (200 MHz, CDCl_3_): *δ* 1.87–1.97 (m, 2H), 2.98 (s, 1H), 3.10 (s, 1H), 3.58–3.66 (m, 3H), 3.86–3.94 (dd,* J* = 2.65, 9.98 Hz, 1H) 4.52 (br s, 2H) 7.25–7.35 (m, 5H); ^13^C NMR (50 MHz): *δ* 32.0, 53.7, 58.5, 61.7, 73.0, 127.6, 128.4, 138.1; Anal. Calcd for C_12_H_16_O_3_: C, 69.21; H, 7.74. Found C, 69.45; H, 7.85.

### 2.3. (R)-5-(Benzyloxy)pentane-1,3-diol, **5**

To a stirred solution of epoxy alcohol (0.15 g, 0.75 mmol) in THF (5 mL) at −15°C dropwise solution of sodium bis(methoxyethoxy)aluminum hydride (Red-al) (3.5 M solution in toluene, 1.2 mmol) was added. The reaction mixture was stirred for 6 h at the same temperature. When no starting material was observed on TLC, the temperature was raised to 0°C, reaction mixture was quenched with citric acid solution, and the resultant reaction mixture was stirred for another 10 min. Then contents were decanted leaving behind a residue, which was further dissolved in water and extracted with EtOAc thrice. The combined organic layers were evaporated under reduced pressure, and the residue was chromatographed over silica gel (60–120 mesh, EtOAc/hexane 3 : 7) yielding pure diol (0.14 g, 96%) as viscous liquid; [α]D25: −5.8 (*c* 0.6, CHCl_3_); IR (neat): *ν* 3447, 3123, 2186, 1769, 1576, 1478, 1267, 1181, 1134, 1096, 748 cm^−1^; ^1^H NMR (200 MHz, CDCl_3_): *δ* 1.65–1.83 (m, 5H), 3.63–3.75 (m, 2H), 3.81–3.86 (m, 2H), 4.04–4.16 (m, 1H), 4.53 (br s, 2H), 7.25–7.35 (m, 5H); ^13^C NMR (200 MHz, CDCl_3_): *δ* 36.5, 38.4, 61.4, 69.0, 71.7, 73.4, 127.8, 128.5, 137.7; Anal. Calcd for C_12_H_18_O_3_: C, 68.54; H, 8.63. Found C, 69.15; H, 7.95.

### 2.4. (R,E)-6-(4-Oxo-6-phenylhex-2-enyl)-5,6-dihydro-2H-pyran-2-one(*R*)-Rugulactone, **1**

Grubb's second generation catalyst (123.1 mg, 0.145 mmol) was added to the stirred solution of lactone (*R*)-**9** (200 mg, 1.45 mmol) and 5-phenylpent-1-ene-3-one** 10** (693.173 mg, 4.347 mmol) in CH_2_Cl_2_; stirring was continued for 12 h at 45°C; when starting material was completely consumed (checked by TLC), the reaction mixture was concentrated and purified by silica gel (100–200 mesh) chromatography (EtOAc/hexane 3 : 7) to yield (*R*)-**1** (293 mg, 75%) as a colorless oil. [α]D25 = −46.2 (*c* 1, CHCl_3_), Lit^5^   [α]D25 = −46.9 (*c* 1, CHCl_3_); IR (neat): *ν* 3067, 2925, 2818, 1720, 1626, 1038, 992, 928, 845, 756 cm^−1^. ^1^H NMR (200 MHz, CDCl_3_): *δ* 2.29–2.36 (m, 2H), 2.59–2.68 (m, 2H), 2.87–2.93 (m, 4H), 4.47–4.61 (m, 1H), 6.01–6.07 (dt,* J* = 1.70, 6.50 Hz, 1H), 6.13–6.23(dt,* J* = 1.49, 14.53 Hz, 1H), 6.71–6.92 (m, 2H), 7.16–7.26 (m, 5H); ^13^C NMR (50 MHz): *δ* 29.0, 30.0, 37.6, 41.8, 121.6, 128.4, 128.5, 133.5, 139.9, 141.0, 144.4, 163.4, 198.6; Anal. Calcd for C_17_H_18_O_3_: C, 75.53; H, 6.71. Found C, 75.46; H, 6.69.

## 3. Results and Discussion

**Scheme 2:**
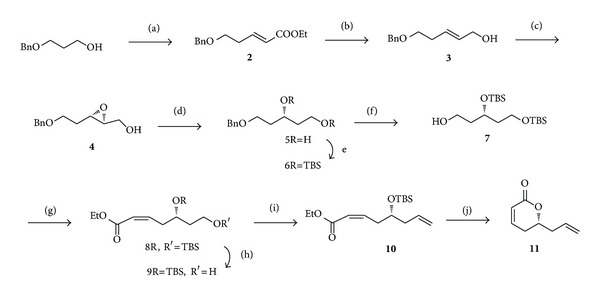
Reagents and conditions: (a) (i) DMSO, (COCl)_2_, Et_3_N, CH_2_Cl_2_, −78°C, 1 h. (ii) Triethyl phosphonoacetate, NaH, dry Benzene, 0°C-RT, 8 h, 95%. (b) LiAlH_4_, AlCl_3_, THF, 0°C,1 h, 82%. (c) (−)-DET, Ti(O-i-Pr)_4_, TBHP, dry CH_2_Cl_2_, molecular sieves 4 Å, −15°C, 87%. (d) Red-al, THF, −20°C, 6 h, 96%. (e) TBDMSCl, Et_3_N, DMAP, CH_2_Cl_2_, RT, 8 h, 96%. (f) Na, Liq. NH_3_, dry THF, −78°C, 15 min, 92%. (g) (i) DMSO, (COCl)_2_, Et_3_N, CH_2_Cl_2_, −78°C, 1 h. (ii) EtO_2_CCH_2_P(O)(OCH_2_CF_3_)_2_, NaH, dry THF, −78°C, 2 h, 74%. (h) CSA, MeOH:CH_2_Cl_2_ (1 : 1), RT, 85%. (i) DMSO, (COCl)_2_, Et_3_N, CH_2_Cl_2_, −78°C, 1 h. (ii) (C_6_H_5_)_3_PCH_3_I, n-BuLi, THF, 0°C, 82%. (j) PTSA, MeOH, 3 h, 91%.

As outlined in Scheme 2, our synthetic strategy commenced with 3-benzyloxypropanol. The primary alcohol of 3-benzyloxypropanol was oxidized by using Swern's protocol to the corresponding aldehyde, and then Horner-Wadsworth-Emmons olefination of aldehyde afforded *α*, *β*-unsaturated ester** 2 **in 95% yield. The compound** 2** was subsequently reduced to allyl alcohol** 3 **by employing alane reduction (LiCl/LiAlH_4_) conditions [29]. The allyl alcohol** 3** was then subjected to Sharpless asymmetric epoxidation [30, 31] to produce epoxy alcohol** 4 **in 85% yield, whichon selective hydride reduction with Red-al [32, 33] yielded 1, 3-diol** 5**. The two hydroxyl groups in** 5 **were completely protected from its disilyl ether** 6**. The subsequent removal of benzyl group was achieved by using Birch debenzylation [34] protocol to afford alcohol** 7**, which was further oxidized to aldehyde and Still-Gennari modification of Horner-Emmons [35] olefination of the crude aldehyde produced Z/E 95 : 5 mixture of *α*, *β*-unsaturated ethyl esters in favor of desired isomer** 8**. The geometric isomers were easily separated using silica gel column chromatography to get pure Z isomer of ethyl ester in 74% yield. Later the primary silyl ether was selectively cleaved to produce alcohol** 9**, which on further oxidation followed by Wittig olefination furnished unsaturated ester** 10**. Further *α*, *β*-unsaturated ester** 10** was stirred in methanol for 2 h in presence of* p*-toluene sulfonic acid to furnish** 11** in 91% yield.

**Scheme 3:**
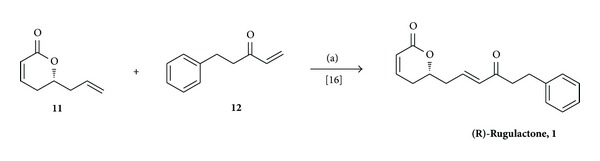
Reagents and conditions: (a) Grubb's 2nd generation catalyst (5 mol %), dry CH_2_Cl_2_, 45°C, 12 h, 75%.

The remaining task was to couple the fragment 5-phenyl-pent-1-en-3-one [36]** 12** and lactone** 11** (3 : 1 ratio) by cross metathesis [37–39], which was implemented by refluxing them in CH_2_Cl_2_ in presence of Grubb's second generation catalyst [40] (5 mol%) to deliver enantiomerically pure (*R*)-rugulactone (**1**) in 74% yield as colorless oil, [α]D25: −46.2 (*c* 1, CHCl_3_), Lit [41][α]D25: −46.9 (*c* 1, CHCl_3_) (see Scheme 3).

## 4. Conclusions

The stereoselective total synthesis of naturally occurring bioactive compound (*R*)-rugulactone has been successfully achieved employing Sharpless asymmetric epoxidation of allyl alcohol, selective hydride reduction of epoxy alcohol, and olefin cross metathesis reactions as the key steps. The synthetic route can conveniently be utilized for the preparation of various analogs of (*R*)-rugulactone useful for biological evaluation.

## Supplementary Material

Full experimental details, ^1^H and ^13^CNMR spectra, can be found in Supplementary Materials.
