# Supplementary material for: A Facile Stereoselective Total Synthesis of (R)-Rugulactone
Source: ISRN Org Chem. 2014 Mar 30;2014:767954. doi: 10.1155/2014/767954 (PMC4041020; doi:10.1155/2014/767954)

A facile stereoselective total synthesis of (*R*)-rugulactone

B. Narasimha Reddy[a] and R.P. Singh[a,b*]

a) Polymer science and engineering division, National Chemical Laboratory, Pashan, Pune 411 008, INDIA
b) Present address: Bharthi vidyapeeth University, Advanced Research Centre in Pharmaceutical Sciences & Applied Chemistry, Poona college of pharmacy, Erandwane, Pune- 411038.
** Corresponding author. Tel: +91-20-25437237 Fax: +91-20-25437237*

E-mail: rp.singh@ncl.res.in

Supporting Information

4.1. General information:

Solvents were purified and dried by standard procedures before use. Optical rotations were measured using sodium D line on a JASCO-181 digital polarimeter. IR spectra were recorded on Thermo Scientific-Nicolet 380 FT-IR Instrument. ^{1}H NMR and ^{13}C NMR spectra were recorded on Brucker AC-200 spectrometer. Elemental analysis was carried out on a Carlo Erba CHNS-O analyzer.

4.8. (S, Z)-ethyl 5, 7-bis(tert-butyldimethylsilyl)oxy)hept-2-enoate, 8

BAIB (4.99 g, 15.51 mmol) and TEMPO (0.162 g, 1.04 mmol) were added to a stirred solution of **7** (1.2 g, 10.34 mmol) in CH_2Cl_2 (30 mL) at room temperature and stirred for 4 h, then reaction was quenched with saturated solution of $Na_2S_2O_3$ in water (0.5 mL). Reaction mixture was diluted with CH_2Cl_2 (30 mL), washed with water and dried over anhydrous Na_2SO_4. The organic layer was concentrated under *vacuum* to get crude aldehyde.

60 % dispersion of NaH (0.448 g, 13.30 mmol) in mineral oil was added to a stirred solution of ethyl P,P bis (2,2,2-trifluoroethyl) phosphonoacetate (3.496 g, 10.52 mmol) in dry THF (50 mL)

at 0 ^{o}C, resulting ylide solution was stirred for 45 min at the same temperature, then the reaction mixture was cooled to -78 ^{o}C. The crude aldehyde obtained above dissolved in dry THF (10 mL) was added drop wise and stirring was continued for further 3 h. After completion of the reaction, the reaction was quenched with saturated NH_4Cl solution (2 mL) at 0 ^{o}C, concentrated under reduced pressure. Residue obtained was dissolved in EtOAc washed with water and brine. Organic layer was dried over Na_2SO_4, evaporated under *vacuum* and the crude product was purified by silica gel column chromatography (100-200 mesh, EtOAc/hexane 2:8) to obtain (*R*)-**8** (1.33 gm, 74%) as colorless oily compound; $[\alpha]_D^{25}$: - 12.3 (*c* 1, $CHCl_3$); IR (neat): ν 3116, 3020, 2922, 1724, 1679, 1326, 1243, 1125, 1141, 1047, 1024, 747 cm^{-1}; ^{1}H NMR (200 MHz, $CDCl_3$): δ 0.12, (br s, 12H), 0.91 (br s, 18H), 1.29-1.33 (t, J = 7.08 Hz, 3H), 1.69 (br s, 1H), 1.75-1.80 (m, 2H), 2.88-2.99 (m, 2H), 3.73-3.85 (m, 2H), 4.12 (m, 1H), 4.16-4.21 (m, 2H). 5.88 (d, J = 11.54 Hz, 1H), 6.38 (m, 1H); ^{13}C NMR (50 MHz, $CDCl_3$): δ -5.4, -4.7, 14.2, 25.7, 40.5, 51.0, 59.2, 60.6, 65.5, 123.4, 145.8, 168.35; Anal. Calcd for $C_{21}H_{44}O_4Si_2$: C, 60.52; H, 10.68. Found C, 61.15; H, 9.98.

4.9. (S, Z)-ethyl 5,7-(tert-butyldimethylsilyl)oxy)-7-hydroxyhept-2-enoate, 9

Camphor sulphonic acid (0.074 g, 0.33 mmol) was added to a stirred solution of compound **8** (0.416 g, 1mmol) in 1:1 mixture of MeOH and CH_2Cl_2 at room temperature. The reaction mixture was stirred for 1.5 h and upon completion; the reaction was quenched with saturated $NaHCO_3$ solution. The product was extracted into CH_2Cl_2, the organic layer was evaporated under reduced pressure and the residue was column chromatographed over silica gel (60-120 mesh, EtOAc/hexane 1:9) to yield pure alcohol **9** (0.054 g, 85 %); $[\alpha]_D^{25}$: + 22.1 (*c* 1, $CHCl_3$); IR (neat): ν 3466, 3116, 3023, 2926, 1732, 1679, 1331, 1267, 1224, 1121, 1098, 945, 743 cm^{-1}; ^{1}H NMR (200 MHz, $CDCl_3$): δ 0.12 (br s, 6H), 0.91(br s, 9H), 1.29-1.33 (t, J = 7.08 Hz, 3H), 1.69

(br s, 1H), 1.75-1.80 (m, 2H), 2.88-2.99 (m, 2H), 3.73-3.85 (m, 2H), 4.12 (m, 1H), 4.16-4.21 (m, 2H), 5.88 (d, J = 11.54 Hz, 1H), 6.38 (m, 1H); ^{13}C NMR (50 MHz, $CDCl_3$): δ -4.8, 14.2, 25.8, 36.1, 38.5, 59.93, 60.1, 70.6, 121.3, 145.9, 166.4 ; Anal. Calcd for $C_{15}H_{30}O_4Si$: C, 59.56; H, 10.00. Found C, 58.79; H, 9.05.

4.10. (R, Z) –ethyl 5-((tert-butyldimethylsilyl)octa-2,7-dienoate, 10

The precursor aldehyde for compound **10** was prepared from the above obtained alcohol **9** following the procedure described under section **4.8**

To a stirred solution of methyltriphenylphosphonium iodide (1.313 g, 3.2 mmol) in THF (10 mL) at 0 ^{o}C, added n- BuLi (1.6 M solution in hexane, 3.2 mmol) drop wise. After stirring for 15 min, a solution of crude aldehyde (0.75 g, 2.5 mmol) in THF (10 mL) was added drop wise. The reaction mixture was stirred at the same temperature for another 2h. Then the reaction was quenched with saturated NH_4Cl, the two layers were separated and the aqueous layer was treated with EtOAc thrice. The combined organic layers were concentrated under reduced pressure to furnish crude product which on further purification by column chromatography over silica gel (60-120 mesh, EtOAc/hexane 1:9) yielded pure compound **10** as color less oil (1.45 g, 85%); $[\alpha]_D^{25}$: - 15.6 (*c* 1, $CHCl_3$); IR (neat): ν 3158, 3024, 2927, 2928, 1737, 1675, 1376, 1279, 1257, 1154, 1136, 1041, 945, 743 cm^{-1}; ^1H NMR (200 MHz, $CDCl_3$): δ 0.12, (br s, 6H), 0.91(br s, 9H), 1.19-1.27 (m, 3H), 2.26–2.37 (m, 2H), 2.80–2.89 (m, 2H), 3.71-3.74 (m, 1H), 4.07-4.21(m, 2H), 5.12–5.19 (m, 2H), 5.04-5.14 (m, 2H), 5.67-5.88 (m, 2H), 6.83-6.98 (dt, J = 3.41, 1H); ^{13}C NMR (50 MHz, $CDCl_3$): δ -4.6, 14.3, 17.2, 25.9, 36.1, 42.0, 59.7, 71.4, 117.2, 121.0, 134.8, 146.4, 166.1; Anal. Calcd for $C_{16}H_{30}O_3Si$: C, 64.38; H, 10.13. Found C, 63.15; H, 10.95.

4.11. (R)-6-Allyl-5, 6-dihydro-2H-pyran-2-one, 11

To a stirred solution of compound **10** (0.594 g, 3 mmol) in MeOH was added catalytic amount of PTSA and the contents were stirred for 3 h at room temperature. When no starting material was observed on TLC, the reaction mixture was concentrated under reduced pressure, dissolved in EtOAc and washed with Na_2CO_3 solution (5 mL, 10%). Organic layer was separated and dried over anhydrous Na_2SO_4, concentrated under *vacuum* and the obtained residue was chromatographed over silica gel (100-200 mesh, CH_2Cl_2/hexane 1:9) yielding (R) - **11** (910 mg, 91%) as a colorless oil; $[\alpha]_D^{25}$: -114.8 (*c* 1, $CHCl_3$); IR (neat): ν 3065, 2921, 2815, 1711, 1615, 1458, 1365, 1241, 1041, 905, 742 cm^{-1}; ^1H NMR (200 MHz, $CDCl_3$): δ 2.34-2.37 (m, 2H), 2.44-2.60 (m, 2H), 4.45-4.52 (p, 1H), 5.15 (s, 1H), 5.17-5.19 (d, 1H), 5.79-5.89 (m, 1H), 6.1-6.03 (d, J = 7.03 Hz, 1H), 6.85-6.90 (m, 1H); ^{13}NMR (200 MHz, $CDCl_3$): δ 28.7, 39.1, 77.0, 118.9, 121.5, 132.3, 144.7, 164.0; Anal. Calcd for $C_8H_{10}O_2$: C, 69.54; H, 7.30. Found C, 69.48; H, 7.19.

^{1}H NMR and ^{13}C NMR of **compound 4** in $CDCl_3$

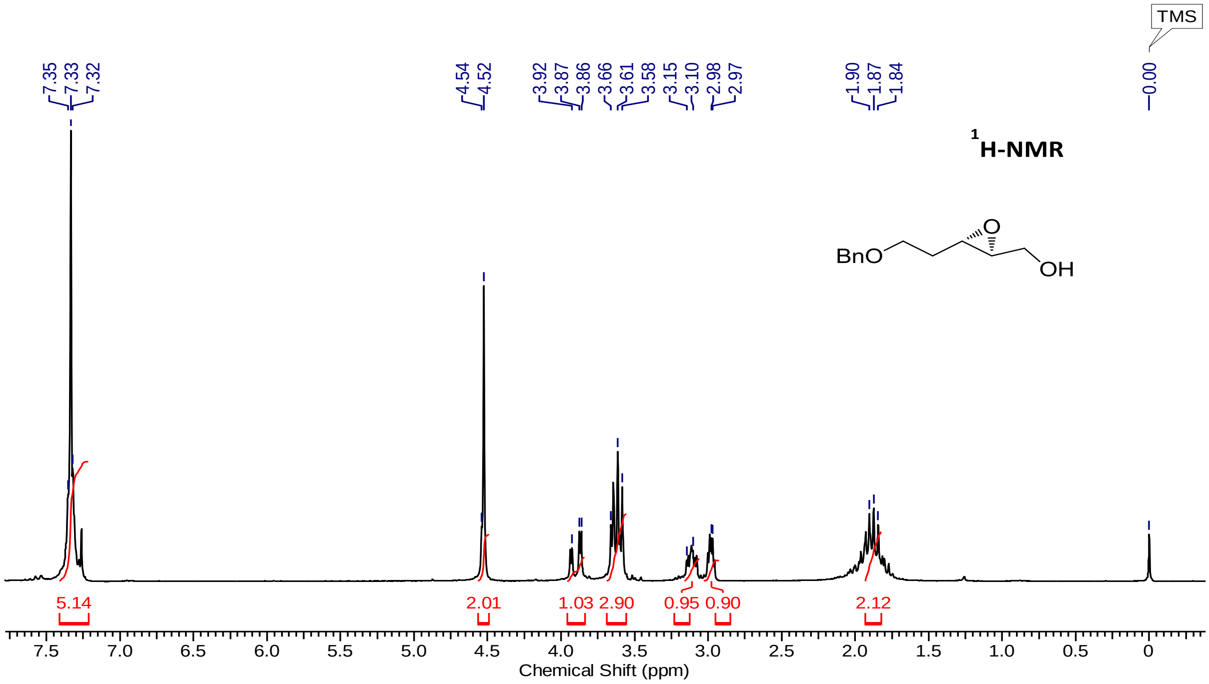

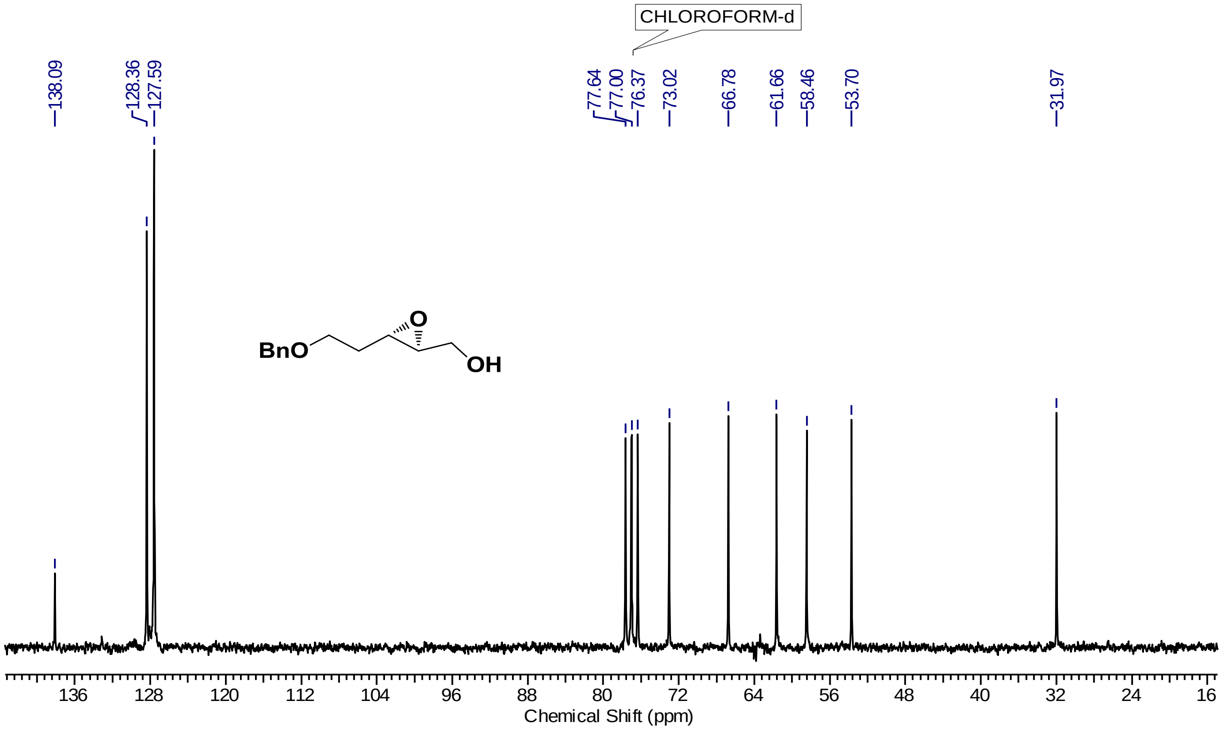

^{1}H NMR and ^{13}C NMR of **compound 11** in $CDCl_3$

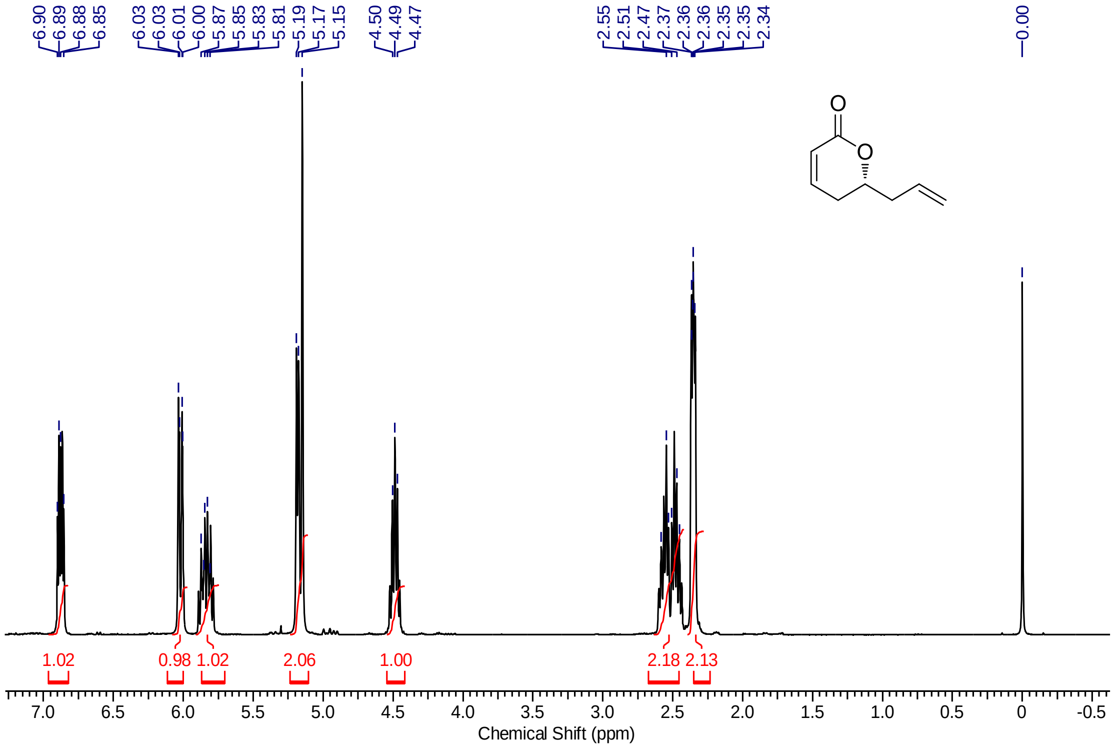

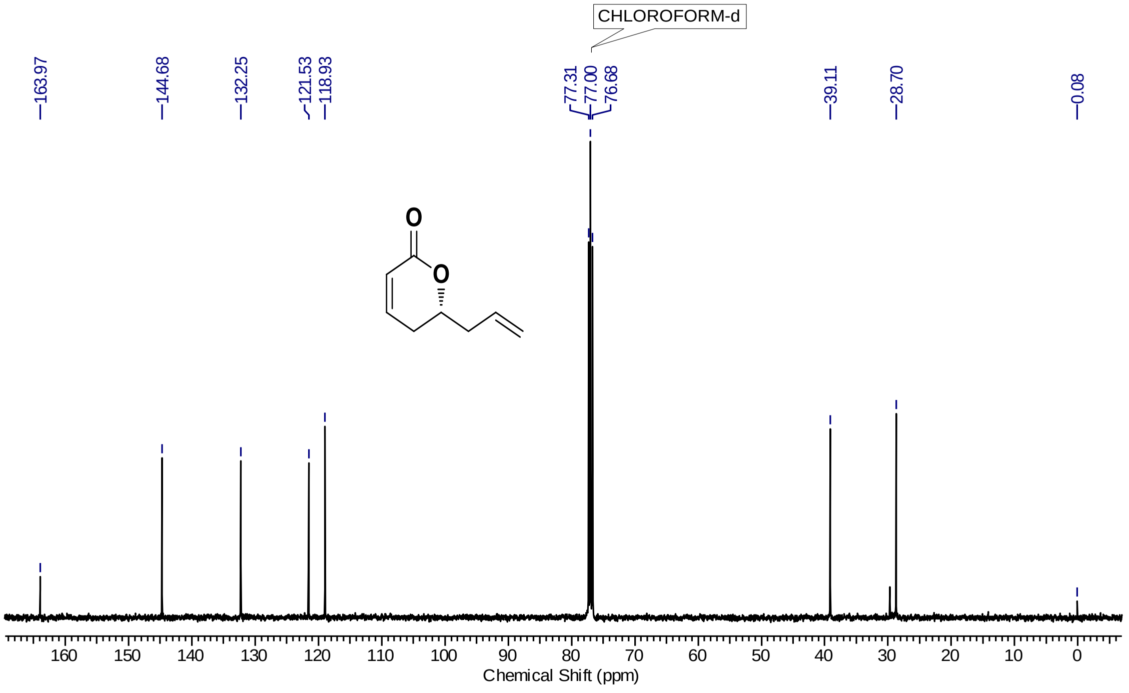

^{1}H NMR and ^{13}C NMR of **compound 1** in $CDCl_3$

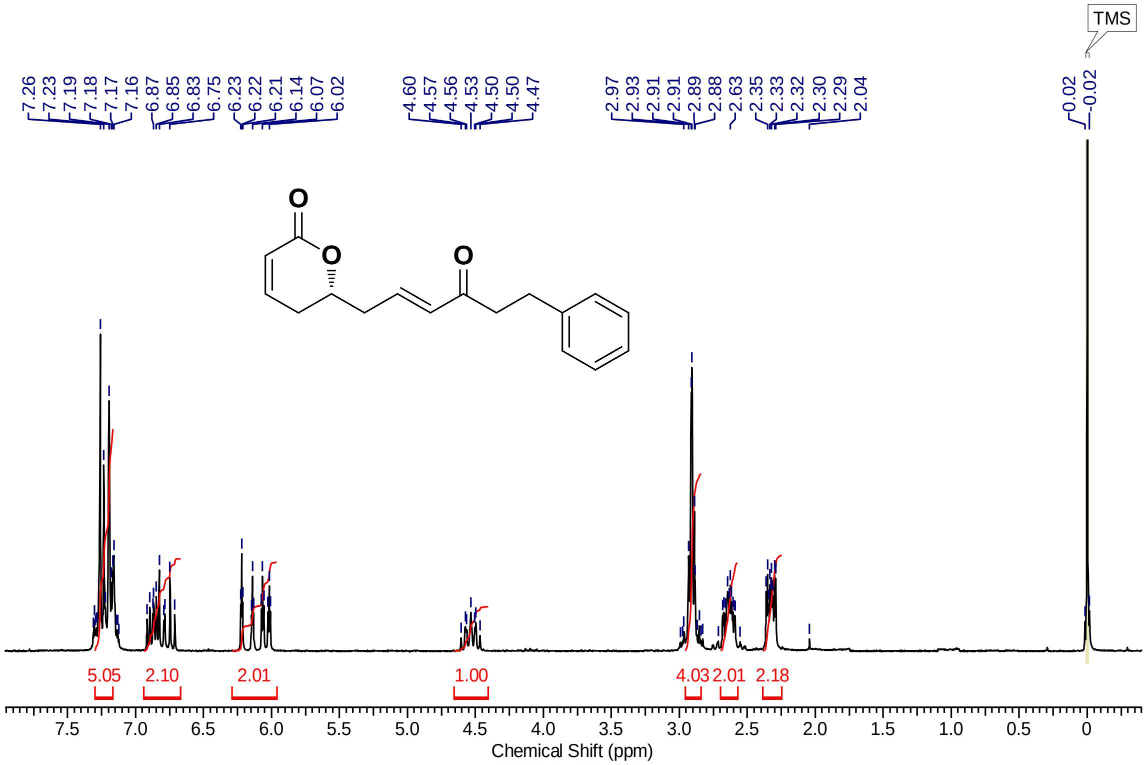

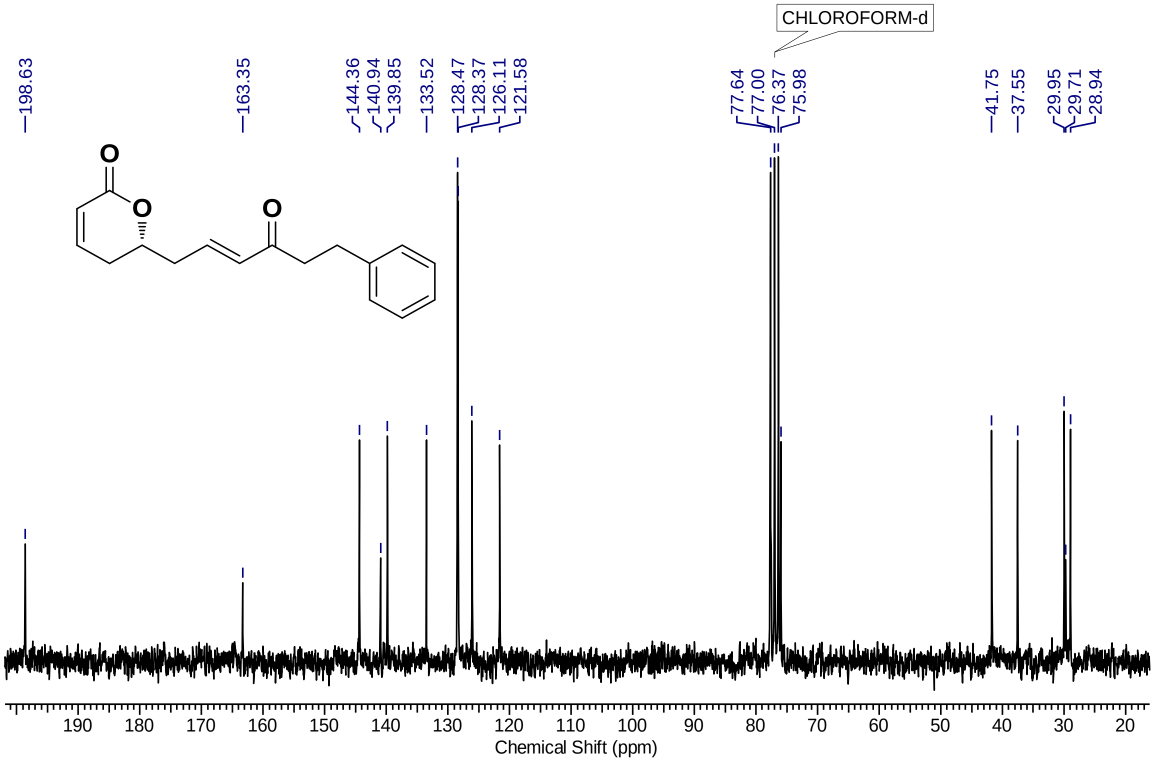

Supplement: Supplementary file 1 — Full experimental details, 1H and 13CNMR spectra, can be found in Supplementary Materials. [file 767954.f1.pdf]
